# Early Disengagement from Gambling Treatment: Insights from Clients and Practitioners

**DOI:** 10.1007/s10899-026-10484-5

**Published:** 2026-03-03

**Authors:** C. O. Hawker, N. A. Dowling, A. C. Thomas, S. S. Merkouris

**Affiliations:** 1 School of Psychology, Deakin University, Burwood, Australia; 2 Melbourne Graduate School of Education, Parkville, Australia

**Keywords:** Gambling, Treatment, Disengagement, Retention, Attendance

## Abstract

Early disengagement may undermine the effectiveness of evidence-based gambling treatment, yet limited research has examined why clients disengage and how services can better support retention. This exploratory mixed-methods study investigated reasons for early disengagement, its potential consequences for clients, and practical strategies to improve retention in real-world gambling services. Data were drawn from a two-timepoint client survey (pre-treatment and one-month follow-up), and a practitioner survey and hui (group discussion), capturing complementary perspectives. Participants were 67 clients (*M*_age_=36.7; 59.7% male; 91.0% with mild-to-extreme gambling symptom severity) and 15 practitioners (*M*_age_=48.3; 20.0% male; 66.7% counsellors) from New Zealand gambling specialist services. Across clients and practitioners, common reasons for early disengagement included logistical barriers, low motivation and external support, co-occurring mental health issues, concern about missing gambling, and gambling-related financial stress. Practitioners also highlighted early goal attainment, non-linear engagement, and systemic barriers. At follow-up, early disengagement was generally associated with making less gains in gambling, psychological, and broader addiction outcomes and greater professional help-seeking for non-gambling issues, compared with clients who completed or remained in treatment. Practitioners noted, however, that even brief engagement can build insight and readiness for future help-seeking. Clients and practitioners identified multi-level strategies to strengthen early retention, centred on relational quality (e.g., empathy, motivational style, peer/group support), proactive contact (e.g., welcome calls, reminders), service flexibility (e.g., multiple delivery formats, after-hours options), and workforce capacity (e.g., culturally responsive training). These findings provide a foundation for future research to evaluate the effectiveness and feasibility of treatment retention strategies in routine care.

## Introduction

Psychological interventions, such as cognitive–behavioural therapy (CBT) and motivational interviewing (MI), are effective treatments for gambling disorder, with 6–8 sessions typically recommended and longer engagement linked to better outcomes (Bodor et al. 2021; Petry et al. 2017; Pfund et al. 2020a, b, 2023). Yet around one-third of clients disengage prematurely, often after only one or two sessions (Hawker et al. 2025a, b, c; Melville et al. 2007; Pfund et al. 2021). New Zealand service data demonstrate a similar pattern to global data, with one-third of clients attending only one session, one-third attending two to five sessions, and one-third attending six or more sessions (Hawker et al. 2025a, b, c). A recent scoping review identified a growing literature on the prevalence, predictors, and definitions of disengagement, but relatively limited and fragmented evidence on its underlying reasons, consequences for clients, or strategies to improve retention, particularly in the early stages of treatment when most clients disengage (Hawker et al. 2025a, b, c).

### Reasons for Early Treatment Disengagement

Few studies have explored reasons for early treatment disengagement. Reasons reported by clients most often include logistical barriers (e.g., scheduling conflicts, work or family commitments), and less often, low motivation, hopelessness, health problems, incarceration, referral elsewhere, and dissatisfaction with treatment (e.g., Carlbring et al., 2010; Dunn et al., 2012; Khanbhai et al., 2017; Maccallum et al., 2007). Broader addictions research identifies similar reasons, including logistical barriers, stigma, and competing demands, (Palmer et al., 2009; Rubenis et al., 2024). In New Zealand, reasons for discharge remain largely unknown as gambling service data record only a pre-determined set of practitioner-recorded discharge reasons (Hawker et al. 2025a, b, c). Research capturing both client and practitioner perspectives is therefore needed to identify modifiable factors that could enhance early treatment retention.

### Consequences of Early Treatment Disengagement

Little is known about the consequences of early disengagement from gambling treatment. Existing findings generally demonstrate that people who complete treatment typically achieve greater improvements in gambling symptom severity, urges, cognitions, and behaviours, as well as psychological distress and depressive symptoms, across various interventions (Battersby et al. 2013; Campos et al. 2023; Grant et al. 2004; Shaffer et al. 2005; Smith et al. 2010a, b, 2015). Interestingly, New Zealand service data indicate that around one-quarter to one-third (24–39%) of clients complete their treatment plan in fewer than six sessions, suggesting that early disengagement may sometimes reflect positive outcomes, such as early goal attainment (Hawker et al. 2025a, b, c). This interpretation is consistent with evidence from brief and single-session gambling interventions, which show that meaningful and sustained improvements can occur even following limited treatment contact (Quilty et al., 2019; Toneatto, 2016). To date, no studies have examined a broad range of consequences of early treatment disengagement.

### Solutions to Improve Treatment Retention

While relatively few studies have explicitly evaluated strategies designed to improve retention in gambling treatment (Hawker et al. 2025a, b, c), many interventions incorporate engagement-enhancing components, including motivational enhancements, involvement of significant others, and concurrent pharmacotherapy (Jiménez-Murcia et al. 2017; Kraus et al. 2020; Nilsson et al. 2020; Pfund et al. 2020a, b). For example, CBT augmented with motivational enhancements has shown higher completion rates than standard CBT by reinforcing attendance and addressing earlier barriers to change (Milton et al., 2002; Wulfert et al., 2006, 2025). Evidence from gambling treatment and the broader addictions literature also points to practical, low-resource strategies to improve attendance, such as role induction, pretreatment contact, and reminder messages (Pfund et al. 2020a, b; Rubenis et al. 2023). Practitioner perspectives further highlight clinician- and service-level factors, such as therapeutic alliance, cultural competence, accessibility, and continuity of care (Palmer et al., 2009; Rubenis et al., 2024). Together, these findings suggest that combining brief motivational and relational strategies with service-level improvements may enhance early treatment retention, underscoring the value of understanding how these approaches are perceived by gambling clients and practitioners.

### The Current Study

A substantial gap remains in understanding the reasons for early treatment disengagement, its consequences for clients, and strategies to improve retention (Hawker et al. 2025a, b, c). This study addressed this gap by exploring client and practitioner perspectives on the reasons, consequences, and potential solutions to early disengagement within gambling harm services in New Zealand. While many drivers of disengagement are likely shared across jurisdictions, New Zealand provides a distinctive service and cultural context, including a publicly funded gambling treatment system and the recognition of several priority populations disproportionately affected by gambling harm (including Māori, Pacific, and Asian peoples), which may shape engagement processes differently from other settings (Ministry of Health, 2025). As jurisdictions require context-specific evidence to strengthen service delivery, findings from this study offer locally relevant insights with broader international implications. Given limited and inconsistent prior findings, analyses were exploratory. Integrating client and practitioner perspectives provides a more holistic understanding of early disengagement and can inform practical strategies to enhance treatment retention and outcomes.

## Method

### Study Design

As part of a broader mixed-methods project evaluating treatment retention within New Zealand gambling services (Hawker et al. 2025a, b, c), this study comprised two components: (1) a prospective, two-timepoint client survey (pre-treatment and one-month follow-up); and (2) a cross-sectional practitioner survey and follow-up ‘hui’ (a Māori term referring to a facilitated group meeting or discussion) to explore service implications of project findings. While recruitment and follow-up were limited by project timelines and ethics-related delays, the one-month follow-up interval was selected to enable preliminary exploration of client experiences during the early treatment period, when most disengagement occurs and intervention may be most effective (Hawker et al. 2025a, b, c; Hawker et al. 2025a, b, c; Pfund et al. 2021).

### Setting

This study was conducted across government-funded outpatient gambling treatment services in New Zealand, which provide free, unlimited treatment for people affected by gambling harm (Ministry of Health, 2025). Treatment varies but typically involves individual psychosocial sessions focused on screening, goal setting, relapse prevention, and exit planning. Although some services recommend up to six sessions over a three-month period, the number, format (in-person or remote), and focus of sessions are tailored to client needs. Clients access services through self-referral or referral from health, social, justice, or community agencies.

### Participants

Eligibility criteria for both samples included being at least 18 years of age, living in New Zealand, and proficiency in English. Clients also had to have recently commenced gambling treatment (within the past month) and attended at least one session by the one-month follow-up, while practitioners had to be currently delivering psychosocial gambling treatment.

The client sample included 67 clients across five services (*M*_age_ = 36.7, SD = 10.7), most of whom were male (59.7%), born in New Zealand (95.5%), and identified as New Zealand European (73.1%). On average, clients scored 26.1 (*SD* = 11.4) on the Gambling Symptom Assessment Scale (G-SAS), corresponding to moderate gambling symptom severity, and 15.6 (*SD* = 6.7) on the Domain General Harms Scale (DGHS-7: Syvertsen et al., 2023). Consistent with this, nearly all clients (97.0%) reported at least one gambling-related harm, most commonly financial (95.5%), emotional (94.0%), health (92.5%), and relationship harms (91.0%).

The practitioner sample completing the survey included 15 practitioners across four services (*M*_age_ = 48.3, *SD* = 13.0), with four practitioners (3 female; 1 male) from two services also participating in the follow-up hui. Most practitioners were female (80.0%) and born in New Zealand (86.7%), with over half identifying as New Zealand European (53.3%) and one-quarter as Māori (26.7%). Practitioners primarily worked in counsellor roles (66.7%) and had an average of 5.6 years (*SD* = 5.1) experience in delivering gambling treatment. Table 1 displays the characteristics of each study sample.

**Table 1:** Background characteristics of the client and practitioner samples

Background characteristics	*n* (%)	
	Client sample (*n* = 67)	Practitioner sample (*n* = 15)
Gender (% male)	40 (59.7)	3 (20.0)
Born in New Zealand	64 (95.5)	13 (86.7)
Ethnicity*		
New Zealand European	49 (73.1)	8 (53.3)
Māori	21 (31.3)	4 (26.7)
Pacific	5 (7.5)	2 (13.3)
Asian	2 (3.0)	1 (6.7)
Other	5 (7.5)	2 (13.3)
Marital status		
Never married	33 (49.3)	-
Married or de facto	21 (31.3)	-
Separated	9 (13.4)	-
Divorced	2 (3.0)	-
Widowed	2 (3.0)	-
Employment status		
Full-time work	33 (49.3)	-
Part-time or casual work	13 (19.4)	-
Full-time or part-time student	5 (7.5)	-
Full-time home duties	7 (10.4)	-
Sick or disability pension	4 (6.0)	-
Unemployed	3 (4.5)	-
Retired	1 (1.5)	-
Other	1 (1.5)	-
Clinical role*		
Counsellor	-	10 (66.7)
Manager	-	4 (26.7)
Peer support worker	-	2 (13.3)
Team leader	-	2 (13.3)
Other	-	1 (6.7)
Gambling-related characteristics		
Gambling Symptom Assessment Scale (G-SAS gambling symptom severity categories)		
Extreme severity (score of 41–48)	7 (10.5)	-
Severe severity (score of 31–40)	16 (23.9)	-
Moderate severity (score of 21–30)	25 (37.3)	-
Mild severity (score of 8–20)	13 (19.4)	-
Non-problem severity (score of 0–7)	6 (9.0)	-
Current or past issue with gambling activities*		
Electronic gaming machines	58 (88.6)	-
Horse/harness/greyhound race betting	21 (31.3)	-
Sports and event result betting	20 (29.9)	-
Number games (e.g., lotteries, keno, bingo)	18 (26.9)	-
Table games (e.g., blackjack, roulette)	16 (23.9)	-
Informal private betting	4 (6.0)	-
Preferred gambling modality		
Combination of venue and online	36 (53.7)	-
Online	19 (28.4)	-
Venue	12 (17.9)	-
Domain General Harm Scale-7 (DGHS-7 gambling harm domains)		
Any financial harms	64 (95.5)	-
Any emotional or psychological harms	63 (94.0)	-
Any health harms	62 (92.5)	-
Any relationship harms	61 (91.0)	-
Any work or study harms	49 (73.1)	-
Any legal harms	34 (50.8)	-
Any cultural harms	32 (47.8)	-
Any gambling harms	65 (97.0)	-

*Participants could endorse multiple responses

### Measures

#### Client Surveys

The client survey captured quantitative and qualitative data across two timepoints (pre-treatment and one-month follow-up) to examine client perspectives on the reasons, consequences, and potential solutions related to early treatment disengagement. A summary of the measures and their psychometric properties is provided in Appendix Table 6.

##### Background Characteristics

The pre-treatment survey collected sociodemographic (age, gender, birth country, ethnicity, marital status, and employment status) and gambling-related (gambling activities of concern, preferred modality, and gambling-related harms (DGHS-7: Syvertsen et al., 2023) characteristics.

##### Reasons for Early Treatment Disengagement

At pre-treatment, perceived barriers to attendance were assessed using the Barriers to Retention Scale (BRS: Zemore et al., 2021), capturing low perceived need for treatment, fear of losing important relationships, concerns about missing gambling, personal limitations (e.g., forgetfulness), and logistical issues (e.g., transport), plus an open-ended item to describe additional barriers. Assessing perceived barriers at pre-treatment enabled identification of anticipated obstacles to engagement that may inform early intervention, regardless of subsequent treatment status.

At follow-up, a series of branching items determined treatment status: (1) *early disengagement* (no upcoming sessions despite not fulfilling allocated sessions or goals or having upcoming sessions but not planning to attend), (2) *completed treatment* (no upcoming sessions due to meeting allocated sessions or goals), or (3) *still in treatment* (upcoming sessions planned to attend). Clients in the *early disengagement* group were then administered the 28-item Reasons for Leaving Treatment Questionnaire (RLTQ: Ball et al., 2006), capturing seven domains (e.g., motivational inconsistencies, program expectations, problem severity), plus an open-ended item to describe additional reasons and clients in the *early disengagement* group were administered an open-ended item to explain their decision not to return to treatment. Although shame and stigma, commonly reported reasons for disengagement, were not assessed as standalone constructs, related processes were captured through items reflecting confidentiality concerns, fear of negative evaluation, and discomfort returning to the service, and could be further elaborated through open-ended responses.

##### Consequences of Early Treatment Disengagement

Consequences of early disengagement were examined via change between repeated (pre-treatment and follow-up) assessments of gambling-related, psychological, social, and help-seeking factors.

##### Gambling-related Factors

Assessments included: gambling symptom severity and urges via the G-SAS (Kim et al., 2009); past-month gambling frequency (number of days gambled) and expenditure (total money lost) via single items; gambling-related cognitions via the Gambling Fallacy subscale of the Jonsson-Abbott Scale (JAS: Jonsson et al., 2017); and gambling urge self-efficacy and importance and priority of change via three sliding scale items (1–10; Dowling et al., 2021).

##### Psychological and Social Factors

Assessments included: wellbeing (spiritual, mental, physical, family) via the Hua Oranga framework (Kingi, 2002); anxiety symptoms via the Generalised Anxiety Disorder Scale (GAD-2: Kroenke et al., 2007); depressive symptoms via the Patient Health Questionnaire (PHQ-2: Kroenke et al., 2003); risky alcohol use via the Alcohol Use Disorders Identification Test-3 (AUDIT-3: Babor et al., 2001); past-month substance use (number of times) via a single-item screener (P. C. Smith et al. 2010a, b); impulsivity via six items from the State Impulsivity Scale (SIS: Iribarren et al., 2011); coping strategies via eight subscales of the Brief COPE (Carver, 1997); and family violence victimisation and perpetration via an adapted Hurt-Insult-Threaten-Scream (HITS) scale (Shirzadi et al., 2020).

##### Help-seeking Behavior

Past-month gambling help-seeking was assessed via an adapted Help-Seeking Questionnaire (HSQ: Rodda et al., 2018) capturing nine support options (e.g., mental health professional or residential facility, land-based exclusion).

##### Solutions To Improve Treatment Retention

At pre-treatment and follow-up, clients were prompted to describe what their practitioner or service could do to support treatment attendance via open-ended items. At follow-up, those in the *‘completed treatment’* or *‘still in treatment’* groups were also asked what helped them remain engaged in treatment.

#### Practitioner Survey and Hui

The practitioner survey collected sociodemographic (age, gender, country of birth, cultural identity) and professional (clinical role, years of experience, primary workplace) information, followed by measures of the perceived reasons, consequences, and potential solutions for early disengagement based on their clinical experience.

##### Reasons for Early Treatment Disengagement

Practitioners described perceived reasons through an open-ended item and rated how often they encountered 12 potential reasons using an adapted RLTQ (Ball et al., 2006) to reflect recent review findings on common reasons (e.g., logistical barriers, hopelessness) (Hawker et al. 2025a, b, c). Shame- and stigma-related processes were explored through items reflecting confidentiality concerns, negative staff interactions, and reluctance to engage with services, and open-ended responses.

##### Consequences of Early Treatment Disengagement

Practitioners described perceived negative consequences of disengagement, as well as perceived gains of even limited engagement, for clients’ gambling, mental health and other addictions, social/family functioning, and likelihood of future re-engagement via open-ended items.

##### Solutions To Improve Gambling Treatment Retention

Practitioners were prompted to describe their top five strategies for improving retention (with at least two required). Strategies could relate to actions taken by practitioners or services more broadly.

##### Practitioner Hui

The follow-up hui involved a semi-structured discussion exploring service implications of project findings, with discussions on reasons, consequences, and potential solutions to disengagement analysed in this study.

### Procedure

Clients were purposively recruited through gambling treatment services that distributed study information to eligible individuals. After providing informed consent, clients completed a 30-minute online pre-treatment survey and were invited to complete a 20-minute follow-up survey one month later (both via *Qualtrics*), with follow-up items tailored to their treatment status (*early disengagement*, *completed treatment*, or *still in treatment*). Up to two email reminders were sent for follow-up completion. Clients received up to 30 pre-treatment; $45 follow-up).

Practitioners were recruited via convenience and snowball sampling to complete a 20-minute online survey (*Qualtrics*) and indicate interest in a follow-up hui. The two-hour hui, hosted by *Zoom* and recorded with verbal consent, presented key project findings (CH), followed by a facilitated discussion on service implications (ND). Practitioners were not reimbursed for the survey but received a $100NZD voucher for hui participation.

The study had ethics approval from the Deakin University Human Research Ethics Committee (2024/HE000278) and Aotearoa Research Ethics Committee (AREC24_63).

### Data Analysis

All data analyses were conducted using Stata v18.0 (StataCorp, 2023). Of 100 clients who completed the pre-treatment survey, 73 completed the follow-up survey (73.0% follow-up rate), and 67 met the eligibility criterion of attending at least one psychosocial session by follow-up. At follow-up, 11 clients were classified in the *early disengagement* group (10 reported no upcoming sessions despite not completing their allocated sessions or goals, and 1 had an upcoming session they did not plan to attend), 16 were in the *completed treatment* group (9 had completed their allocated sessions, and 7 had not completed all sessions but had met their goals), and 40 were *still in treatment* (had upcoming sessions they planned to attend). Client and practitioner survey data was complete due to forced survey responses. Fisher’s exact tests were used to examine differences in sociodemographic characteristics by treatment status (early disengagement vs. completed/still in treatment), including gender (male vs. female), ethnicity (New Zealand European vs. other), employment status (full-time or part-time work vs. other), and marital status (married/de facto vs. other), due to small cell sizes and unequal group distributions.

Quantitative data were summarised using descriptive statistics (means, standard deviations, frequencies, percentages). In addition, client-reported consequences were analysed using non-parametric tests given the small sample size and non-normal distribution. Given the small number of clients who disengaged early (*n* = 11), all inferential analyses comparing treatment status were considered exploratory and descriptive in nature, intended to identify patterns of association rather than to support definitive hypothesis testing. Specifically, Spearman correlations (significance set at *p* <.05) were used to explore associations between treatment status (ref: early disengagement vs. a combined group of completed/still in treatment) and percent change scores in repeated measures. Correlation strength was interpreted as negligible (rs= 0.00 to ± 0.20), weak (± 0.21 to ± 0.40), moderate (± 0.41 to ± 0.60), strong (± 0.61 to 0.80), and very strong (± 0.81 to ± 1.00) (Prion & Haerling, 2014). Given the exploratory nature of the study in which analyses were not designed to test a priori hypotheses, no formal power analyses were conducted. This study aimed to recruit as many participants as possible within the project timeframe.

Qualitative data from open-ended client and practitioner survey items and the hui were analysed using a qualitative descriptive approach, which is well suited to summarising participant perspectives in accessible language while staying close to participants’ own words (Colorafi & Evans, 2016; Sandelowski, 2000). Analysis was primarily inductive and pragmatic, with responses grouped into descriptive categories within each topic area. One researcher (CH) reviewed all responses, developed preliminary categories, and refined these in consultation with the research team to ensure categories were comprehensive and reflected the range of participant views. Formal inter-rater coding was not conducted, as the aim was descriptive synthesis rather than theory generation. For the solutions component, findings across clients and practitioners were integrated to identify multi-level implementation strategies (client, practitioner, service, policy) to support treatment retention.

## Results

### Descriptive Statistics

By the 1-month follow-up, clients attended an average of 3.6 sessions (SD = 3.1). Most clients saw a counsellor (88.1%) and received face-to-face (49.3%), remote (26.9%), or hybrid (26.9%) treatment. Clients in the *early disengagement* group (*n* = 11) attended an average of 2.5 sessions (SD = 1.9), with over half (54.6%) attending only one session; while clients who had *completed treatment* (*n* = 16) attended an average of 3.5 sessions (*SD* = 3.1), and those *still in treatment* (*n* = 40) had attended 3.9 sessions (*SD* = 3.3). Fisher’s exact tests indicated no significant differences between clients who disengaged early and those who completed or remained in treatment with respect to gender, ethnicity, employment status, or marital status (all *p* >.18).

### Reasons for Early Treatment Disengagement

#### Client Perspectives

At pre-treatment, clients reported relatively low-to-moderate endorsement of perceived barriers to attendance. The most common perceived barrier was concern about missing gambling (*M* = 3.8), followed by logistical barriers (*M* = 3.2), personal limitations (e.g., forgetfulness; *M* = 3.1), low perceived need for treatment (*M* = 2.5), and fear of losing important relationships (*M* = 2.2). In open-ended responses, clients most often described logistical barriers related to work and family commitments and scheduling conflicts. Several clients described gambling and mental health difficulties as intertwined obstacles, particularly in relation to a perceived need to gamble to manage or escape financial stress. Clients also mentioned emotional or contextual barriers such as embarrassment, incarceration, physical health problems, or practitioner-related concerns, including a preference for a female therapist due to their trauma history.

At follow-up, ten clients who disengaged early (indicated by having no upcoming sessions despite not fulfilling their allocated sessions or goals) completed the RLTQ (see Table 2) to articulate on reasons for disengagement from treatment. The most common reasons reported were severity of gambling or mental health issues (*n* = 8; 80.0%), logistical problems (*n* = 7; 70.0%), motivational challenges (*n* = 6; 60.0%), and outside influences (*n* = 6; 60.0%), with the most common item endorsed being “*mental health or psychological problems kept me from coming*” (*n* = 7). In open-ended responses, individual clients reported embarrassment, a lack of service follow-up, and a mismatch between their needs and the treatment approach: *“the focus of the treatment was more for long term. It did not help me resolve my short-term problems”* (Male, 26). The remaining client who disengaged early attributed this decision to an early treatment focus on financial stress (“*efforts are too focused on short term urgent debt*,* it’s not allowing me to sleep”*, Male, 28). These findings suggest that mental health, logistical, motivational, and external factors emerged as key reasons for early client disengagement.

**Table 2:** Client-reported reasons for early treatment disengagement

Reason for early treatment disengagement	
Barriers to Retention Scale (reported at pre-treatment; *n* = 73)	***M*** **(*SD*)**
Concerns about missing gambling	3.8 (2.1)
Logistical barriers (e.g., transportation, scheduling issues)	3.2 (1.5)
Personal limitations (e.g., tendency to forget things)	3.1 (1.4)
Low perceived need for treatment	2.5 (1.5)
Social concerns (e.g., lose relationships)	2.2 (1.6)
Reasons for Leaving Treatment Questionnaire (reported at follow-up; *n* = 10)	***n*** **(%)**
Problem severity	8 (80.0)
My medical problems kept me from coming in.	0 (0.0)
My gambling was so frequent I could not come in.	4 (40.0)
My drug and/or alcohol use was so heavy I could not come in.	2 (20.0)
My mental health or psychological problems kept me from coming.	7 (70.0)
Logistical problems	7 (70.0)
I had transportation problems that kept me from coming.	3 (30.0)
I had childcare problems that kept me from coming in.	3 (30.0)
The hours of the service were not good for me.	1 (10.0)
I did not have money or insurance to pay for the service.	3 (30.0)
Motivational inconsistencies	6 (60.0)
I changed my mind about being in treatment at this point.	4 (40.0)
I had no good reason to stop gambling.	0 (0.0)
I did not feel motivated enough to keep coming.	4 (40.0)
I lost hope in my ability to change right now.	6 (60.0)
Outside influences	6 (60.0)
Problems with family or acquaintances kept me from coming in.	2 (20.0)
I felt that I could get better on my own or with self-help meetings.	2 (20.0)
I did not have enough support from people in my life to stay at the service.	3 (30.00)
I decided to go to another program for help.	1 (10.0)
Boundary concerns	2 (20.0)
I felt my privacy or confidentiality might not be protected.	1 (10.0)
Somebody I know is a client or staff at the service.	0 (0.0)
I said or did some things that would make it hard for me to go back.	0 (0.0)
I was worried I would get involved in things like gambling, drugs, sex, or crime because of people around the service.	1 (10.0)
Program expectations	2 (20.0)
I did not like the rules of the program.	1 (10.0)
I was confused about what the service wanted me to do.	0 (0.0)
I did not like the kind of services on offer.	2 (20.0)
The wait to start the counselling sessions was too long.	0 (0.0)
Staff conflicts	1 (10.0)
I had a negative interaction with another client or staff.	0 (0.0)
I did not like or trust some of the staff.	1 (10.0)
I felt that staff did not like, respect, or want to help me.	0 (0.0)
I had a personality conflict with people at the service.	0 (0.00)

*M* = mean; *SD* = standard deviation. Barrier to Retention Scale items were rated 1–7, with higher scores indicating greater perceived barriers. Reasons for Leaving Treatment Questionnaire items (yes/no) have been ordered from most to least frequently reported

#### Practitioner Perspectives

In the online survey, practitioners most often attributed disengagement to early goal attainment (*M* = 4.1) or, conversely, low motivation (*M* = 3.4), hopelessness (*M* = 3.3), limited support (*M* = 3.3), a lack of progress (*M* = 3.0), worsening problems (*M* = 2.8), and logistical barriers (*M* = 2.5; see Table 3). Open-ended responses emphasised fluctuating motivation and readiness to change, with many clients observed as cycling in and out of treatment. Stigma and shame, particularly after relapse, were also common barriers. Conversely, some disengaged because their goals were met, with brief contact viewed as sufficient: “*For some*,* just making the phone call can get them back on track” (Counsellor)*.

Insights from the practitioner hui (*n* = 4) reinforced survey findings and highlighted additional contextual and relational factors. Additional factors discussed at the hui included that disengagement often mirrored clients’ initial reasons for seeking help, such as fulfilling external requirements to revoke self-exclusion orders, poor client-practitioner fit, staff changes due to leave or turnover, and limited service options, particularly for culturally or LGBTIQA+ affirming care: *“We are such a small resource; if there isn’t a fit*,* we don’t have all those options available” (Manager)*. Overall, findings suggest that while disengagement often stems from client-level factors (e.g., motivation and life pressures), systemic and relational issues (e.g., continuity of care, fit, workforce capacity) may also play a key role.

**Table 3:** Practitioner-reported reasons for treatment disengagement (*n* = 15)

Reasons for gambling treatment disengagement	M (SD)
Reached treatment goals early or got what they needed from treatment	4.1 (0.9)
Low motivation or commitment to change	3.4 (1.1)
Feelings of hopelessness	3.3 (1.2)
Limited support from others	3.3 (1.4)
Lack of treatment progress	3.0 (1.1)
Worsened problem severity kept client from coming in	2.8 (0.9)
Logistical problems (e.g., transportation, childcare)	2.5 (0.7)
Did not like the services on offer	2.2 (1.2)
Referred or transferred to a different service	2.1 (1.1)
Negative interactions with, or distrust of, staff	2.0 (1.1)
Privacy or confidentiality concerns	1.7 (0.9)

*M* = mean, *SD* = standard deviation. Items were rated on a 5-point scale from 1 (not at all often) to 5 (extremely often). Reasons are ordered from most to least frequently encountered in clinical practice based on mean ratings

### Consequences of Early Treatment Disengagement

#### Client Perspectives

In these exploratory analyses, negative correlations suggested that clients who disengaged early (*n* = 11) generally made less gains across multiple outcomes than those who completed or remained in treatment (*n* = 56). Early disengagement was weakly-to-moderately associated with less gains in gambling symptom severity (*r* = −.40, *p* =.001), gambling urges (*r* = −.37, *p* =.002), past-month gambling frequency (*r* = −.26, *p* =.036), anxiety symptoms (*r* = −.36, *p* =.002), depressive symptoms (*r* = −.38, *p* =.005), risky alcohol use (*r* = −.25, *p* =.036), past-month substance use (*r* = −.26, *p* =.033), and help-seeking for non-gambling mental health issues (*r* = −.25, *p* =.036). The positive correlation for gambling urge self-efficacy (*r* =.30, *p* =.013) reflects that improvement on this measure is scored in the opposite direction; thus, early disengagement was also associated with less gains in self-efficacy. There were no significant associations between treatment status and change in gambling expenditure, cognitions, importance or priority of change, wellbeing, family violence, impulsivity, or coping strategies (see Appendix Table 7).

#### Practitioner Perspectives

In the online survey, practitioners predominantly described negative consequences of disengagement (see Table 4), including an exacerbation of gambling behaviour and harms, worsened mental health and other addictions, and increased isolation or mistrust of services. Some practitioners also observed that even limited engagement could offer meaningful gains, such as insight, autonomy, and readiness for future help-seeking: *“Even one or two sessions can provide insight… and could plant some seeds and encourage engagement in the future.” (Manager/Counsellor).*

During the hui, practitioners further emphasised that limited attendance does not always signal poor outcomes, and instead advocated for broader, culturally grounded indicators of recovery capital, such as connection, purpose, and relational wellbeing: *“You can get a lower PGSI (measure of problem gambling severity) but still have no housing*,* still have lots of debt… you really need to get a bigger picture” (Manager).* They stressed the need for outcome frameworks reflecting diverse recovery goals, including co-occurring issues, relational healing, and culturally defined wellbeing.

**Table 4:** Practitioner-reported consequences of treatment disengagement

	Perceived gains of limited engagement	Perceived negative consequences of disengagement
Gambling behaviour and issues	• Gained insight into their gambling behaviour and patterns • May have achieved their goals, decided to stop gambling, and successfully done so • Had the opportunity to apply strategies and engage in self-management	• Continued or worsened cycle of gambling • Reinforced unhelpful beliefs, and reduced confidence, in ability to change • Stronger gambling urges and greater financial losses • Heightened vulnerability to gambling-related harms
Mental health and other addictive issues	• Gained insight into the connection between gambling and mental health • Clarified the changes needed to improve wellbeing or reach their goals	• Deterioration in mental health, anxiety, depression, hopelessness, substance use, and self-esteem • Increased risk of harm to self or others • Reduced quality of life due to difficulty covering essential expenses
Social and family functioning	• Opportunity to move forward and support others through shared experience	• Greater social withdrawal and isolation • Loss of support from family or friends due to strain or frustration • Increased risk of family conflict or violence associated with ongoing distress
Likelihood of engaging in future treatment	• Increased awareness of available services • Early sessions may encourage future re-engagement	• Decreased willingness or delayed return to treatment, especially after negative treatment experiences • Loss of confidence in treatment effectiveness

### Potential Solutions To Improve Treatment Retention

Client and practitioner data from open-ended survey questions and the subsequent hui identified consistent themes around relational quality, service flexibility, and cultural responsiveness, supported by an adequately resourced system.

#### Client Perspectives

Clients identified strategies to improve retention that spanned four themes: supportive relationships, flexible services, consistent contact, and peer or group support.

***Supportive therapeutic relationships***. Clients viewed the therapeutic relationship as central to ongoing engagement. Warmth, empathy, and encouragement were highly valued, particularly when practitioners helped them gain insight into gambling and underlying issues: *“My counsellor has been very supportive and encouraging*,* keeps me focused”* (Female, 68). Proactive early contact also strengthened engagement: *“A call first to introduce themselves or email”* (Female, 31).

***Flexible and accessible services***. Clients discussed the importance of service flexibility to manage competing demands. Online and hybrid delivery were positively perceived for convenience: *“Convenient that I can attend through Zoom… also attend women’s support group”* (Female, 68); as well as after-hours sessions, more delivery options, and clearer communication about treatment pathways: “*Give me options of what would be beneficial for me”* (Male, 26).

***Ongoing support and incentives***. Regular follow-ups were seen as vital for maintaining engagement: *“Reaching out weekly to see how people are tracking”* (Male, 33). Family or peer support also aided accountability for the person gambling. Some people suggested incentives to sustain engagement: *“Incentives for motivation because the root of gambling is the incentive to get a monetary reward”* (Male, 38).

***Peer and group support***. Many clients suggested that involvement in group or peer-based supports would improve attendance by encouraging shared learning and connection: *“Start a group thing… talk about why you gambled and why you can’t stop”* (Male, 19); *“Introduce me to others… who can find other ways to connect over things except gambling”* (Female, 49).

#### Practitioner Perspectives

Practitioners identified strategies to improve retention that spanned three themes: supportive and client-centred relationships, flexible and accessible delivery, and culturally responsive, well-resourced systems.

***Supportive and client-centred relationships***. Practitioners stressed that attendance improves when clients feel understood and supported. Empathy, trust, and flexibility to address co-occurring issues were viewed as essential: “*Counselling might be the only point of face-to-face contact they get”* (Counsellor). They also highlighted the value of motivational interviewing, collaborative goals, and the option to change practitioners when the fit was poor.

***Flexible and accessible service delivery***. Flexibility was seen as key to overcoming time, transport, and motivational barriers. Practitioners recommended stepped-care models, after-hours scheduling, multiple delivery formats, and simple reminder systems: *“Consider all barriers to help-seeking and design the service to reduce these… matching the right service*,* at the right intensity*,* at the right time”* (Manager). Using more welcoming language (e.g., avoiding the term *“treatment”*) was also suggested to reduce anxiety: *“People are nervous*,* don’t know what to expect”* (Counsellor).

***Culturally responsive and well-resourced systems***. Practitioners emphasised the need for a diverse, supported workforce to deliver culturally grounded, identity-affirming care. Greater investment in training, supervision, and workforce capacity was prioritised: *“There is more available to train an AOD practitioner than a gambling practitioner”* (Manager). Increasing staffing, reducing waitlists, and supporting practitioner wellbeing were also viewed as essential.

Collectively, clients and practitioners agreed that improving attendance requires empathetic, flexible, and culturally informed care, underpinned by a well-resourced workforce and system able to meet diverse client needs.

#### Multi-level Implementation Strategies

Study findings were subsequently integrated to generate a set of implementation strategies across four levels (policy, service, practitioner, and client), summarised in Table 5.

**Table 5:** Client- and practitioner-reported strategies to improve gambling treatment attendance mapped across levels of action

Level of action	Strategies to support attendance in gambling treatment
Policy/Sector	• Fund sustainable, diverse, and specialised gambling workforce development • Expand gambling-specific education, placement, and professional training opportunities • Resource stepped-care, digital, and outreach models • Support complementary non-clinical roles and holistic frameworks within funded service models • Improve coordination across gambling, broader addiction, and mental-health sectors • Promote public awareness and destigmatising language around help-seeking
Service	• Provide flexible scheduling (after-hours, weekends) and multiple delivery formats (individual, group, peer, online) • Maintain continuity of care during staff leave or turnover • Implement reminder systems and simple booking processes • Offer stepped-care pathways from self-help to intensive support • Create clear, inviting communication to reduce anxiety • Increase staffing to reduce waitlists and ensure coverage • Support ongoing professional development and supervision
Practitioner	• Establish warm, empathic, and consistent relationships • Use motivational interviewing and client-led goal setting • Provide proactive outreach (welcome calls, reminders, brief check-ins between sessions) • Offer flexibility in content, location, and mode of delivery • Ensure good client-practitioner fit and support changes as needed • Deliver culturally responsive and trauma-informed care • Address co-occurring issues within a “one-stop-shop” approach, which may involve collaborating with other providers to meet practical and wellbeing needs
Client	• Engage actively in sessions and practise strategies between appointments to strengthen personal coping and self-management (e.g., urge control, distraction, self-exclusion) • Build and use family and peer supports for accountability

Strategies reflect combined insights from clients (pre-treatment and follow-up) and practitioners (survey and hui). Actions are grouped by the level at which they can most feasibly be implemented, recognising shared responsibility across the policy/sector, services, practitioners, and clients

## Discussion

Approximately one-third of clients disengage from gambling treatment, often after only one or two sessions, potentially undermining the benefits of evidence-based care (Hawker et al. 2025a, b, c; Melville et al. 2007; Pfund et al. 2020a, b, 2021). This mixed-methods study extends prior research by exploring reasons for early disengagement, its consequences for clients, and strategies to improve retention across client and practitioner perspectives within specialist gambling services. The findings identified intersecting individual, relational, and systemic factors that shape early disengagement and inform practical, multi-level strategies that may enhance treatment retention.

### Reasons for Early Treatment Disengagement

Building on limited prior research that has primarily identified logistical and motivational reasons for early disengagement (e.g., Carlbring et al., 2010; Khanbhai et al., 2017; Maccallum et al., 2007), this study identified a broad range of practical, psychological, and systemic reasons. Clients and practitioners cited logistical barriers (e.g., scheduling conflicts, competing demands), alongside low motivation, limited external support, and co-occurring mental health issues. Clients also described concern about missing gambling and feeling compelled to gamble to manage financial stress, creating a cycle where distress and debt both sustained gambling and reduced attendance. Client-identified service-related issues, including limited follow-up, poor goal alignment, and overwhelming debt-focused discussions, were identified as further contributing to early disengagement. Practitioners similarly noted that clients often cycle in and out of treatment, reflecting the non-linear nature of engagement shaped by fluctuating readiness, competing priorities, and systemic issues, including workforce shortages and limited access to culturally or LGBTIQA+ responsive care.

Notably, practitioners often attributed early disengagement to goal attainment, whereas clients rarely cited meeting their goals as a reason for not returning. This discrepancy may reflect differing interpretations of ‘treatment completion’, with some clients achieving meaningful progress without recognising or reporting it as goal attainment. Consistent with client accounts, this may partly reflect embarrassment, limited follow-up, or a mismatch between treatment focus and immediate needs, particularly when early treatment discussions emphasise financial stress or longer-term goals, potentially obscuring incremental or non-financial gains.

In this study, around one in ten clients (10.4%) met their goals before completing their allocated sessions, suggesting that meaningful progress can occur earlier than expected, albeit based on a small sample (7 of 67 clients). This pattern aligns with service data showing that a proportion of clients improve within relatively few sessions; for example, one-third of New Zealand clients (23.7%–38.8%) complete treatment in fewer than six sessions (*n* = 18,642; Hawker et al. 2025a, b, c) and half of U.S. clients fully or substantially reach their goals in as little as 5 weeks of exposure-focused CBT (*n* = 410; Khanbhai et al., 2017). Future research should routinely collect goal-related data to distinguish constructive (goal-based) from problematic (barrier-based) disengagement and identify modifiable barriers to treatment retention.

### Consequences of Early Treatment Disengagement

This study identified several potential consequences of early disengagement across gambling, psychological, and help-seeking domains. Compared to clients who completed or remained in treatment at the one-month follow-up, those who disengaged early made less gains in gambling symptom severity, urges, frequency, confidence in managing urges, and depressive symptoms, consistent with limited prior research (Campos et al. 2023; Grant et al. 2004; Smith et al. 2010a, b, 2015). Extending this evidence, early disengagement was also associated with smaller gains in anxiety symptoms, risky alcohol use, and substance use, as well as greater professional help-seeking for non-gambling mental health issues. Importantly, these findings should be interpreted cautiously given the small sample who disengaged early, rendering observed associations sensitive to individual cases. These results tentatively suggest that leaving treatment prematurely may constrain the broader psychological and behavioural benefits of engagement. Practitioners’ perspectives echoed these findings, with most perceiving that early disengagement exacerbates gambling and related harms, worsens mental health, and erodes trust in gambling services.

Several practitioners, however, emphasised that even limited contact can foster insight, autonomy, and motivation for future help-seeking. Hui discussions further underscored the need to broaden definitions of treatment success, recognising that clients may achieve gains in connection or purpose even without full symptom remission. These insights align with the concept of ‘recovery capital’, defined as the internal and external resources supporting sustained recovery (Gavriel-Fried, 2018; Gavriel-Fried & Lev-El, 2020). Practitioners noted that disengagement can limit the development of human and social recovery capital (e.g., coping skills, supportive relationships) but acknowledged that some clients draw on community or cultural strengths to maintain change outside formal treatment. Future research should examine how treatment engagement contributes to the development of recovery capital across a broad range of domains.

### Solutions to Improve Gambling Treatment Retention

Given the relatively small and methodologically diverse body of evidence on strategies to improve retention in gambling treatment (Milton et al., 2002; Wulfert et al., 2006), this study offers novel insights from both clients and practitioners, highlighting converging themes around relational quality, service flexibility, and system-level support. Across client and practitioner perspectives, the therapeutic relationship was viewed as central to sustaining attendance, consistent with broader addictions research linking warmth, empathy, and trust with greater engagement and motivation (Meier et al., 2005; Rubenis et al., 2024). Clients described how supportive relationships helped them persist through setbacks, while practitioners underscored the value of motivational interviewing, collaborative goal setting, and flexibility to address co-occurring issues. These findings position relational quality not only as a foundation for treatment but as an active mechanism for retention.

Both clients and practitioners emphasised the importance of proactive and flexible engagement strategies. Clients valued early contact and consistent follow-up, echoing evidence that welcome calls, reminders, and motivational messages improve attendance (Pfund et al. 2020a, b; Rubenis et al. 2023). Flexibility in delivery (e.g., online/hybrid formats, after-hours appointments) was also viewed by both groups as critical for overcoming logistical and motivational barriers, consistent with stepped-care and person-centred approaches that improve engagement (Park et al., 2021; Rodda, 2024). Clients further identified peer and group support as underused but desirable options for fostering accountability, belonging, and sustained motivation (Hutchison et al., 2018; Penfold & Ogden, 2024). Practitioners noted that these strategies depend on an adequately resourced, culturally responsive workforce capable of delivering identity-affirming care (Abbott, 2020; Palmer et al., 2009). Future research should examine the feasibility and effectiveness of implementing these strategies in routine gambling treatment.

### Practice Implications

This study offers practice-relevant strategies grounded in client and practitioner perspectives to improve early retention in gambling treatment (see Table 5). The findings provide a call to action for policymakers, services, practitioners, and clients to strengthen early engagement and reduce avoidable disengagement. At the policy and sector level, sustained investment in workforce development, supervision, and culturally specific training is needed to ensure equitable access and stronger coordination across gambling, mental health, and financial counselling services. Services play a crucial role by incorporating after-hours scheduling, multiple delivery formats, reliable follow-up systems, and destigmatising communication to reduce barriers to help-seeking. Practitioners can further promote retention through warm, empathic relationships, motivational techniques, proactive outreach, and flexibility to address mental health and financial issues. At the client level, strengthening early motivation, practicing skills between sessions, and involving family or peer supports can build accountability and readiness to change. Collectively, these findings highlight that treatment engagement is a shared responsibility across systems, services, practitioners, and clients. When implemented together, these strategies provide a practical framework for improving continuity of care and recovery outcomes for people experiencing gambling harm.

### Study Limitations

This study has several limitations. Client recruitment and follow-up were constrained by project timelines and ethics-related delays, resulting in small sample sizes and only eleven clients classified in the early disengagement group, which limited both the depth of exploration and the ability to examine longer-term treatment processes. As a result, quantitative analyses comparing early disengagement with continued or completed treatment were exploratory and descriptive in nature; effect estimates may be unstable and sensitive to individual observations, and findings should be interpreted as indicative patterns rather than precise estimates. No formal power analyses were conducted, as the study aimed to recruit as many participants as possible within the project timeframe and was not designed to test a priori hypotheses. Nevertheless, the one-month follow-up provided valuable insight into early treatment experiences, when most disengagement occurs (Hawker et al. 2025a, b, c; Pfund et al. 2021). All data were self-reported and largely descriptive, limiting causal interpretation. While the hui offered rich qualitative data, qualitative survey insights were based on brief open-ended responses. The qualitative analysis prioritised descriptive synthesis over interpretive depth, which may limit theoretical insight but aligns with the study’s applied aims. The small New Zealand workforce also restricted practitioner participation, and participants may not represent less-engaged clients or practitioners. Notably, this study was conducted within New Zealand’s government-funded gambling services, where free, unlimited treatment likely reduces financial barriers to attendance (Abbott, 2020; Ministry of Health, 2025). Future studies would benefit from larger and more diverse samples, extended follow-up, and richer qualitative interview data from clients who disengage from treatment to capture longer-term and culturally specific experiences.

## Conclusion

This study provides new evidence that individual, relational, and systemic factors intersect to influence early treatment disengagement, with consequences extending beyond gambling to broader mental health and addictive behaviours. By integrating client and practitioner perspectives, the study identifies practice-relevant strategies to improve retention, centred on relational quality, proactive contact, service flexibility, and workforce capacity. Embedding these strategies across policy/sector, service, practitioner, and client levels may strengthen continuity of care and recovery outcomes among people seeking treatment for gambling harm.

## Appendix

**Table 6:** Description and psychometric properties of client survey measures (in order of presentation in the manuscript)

Construct	Measure description
**Client survey measures**	
Background characteristics	A series of single items collected sociodemographic characteristics including age (in years), gender (male, female, trans or gender diverse, non-binary, prefer not to specify, prefer to self-describe, other), birth country (New Zealand, other), ethnicity (Māori, New Zealand European, Pacific, Asian, other), employment status (work full time, work part-time/casual, full-time student, full-time home duties, retired, sick or disability pension, other), and marital status (never married, widowed, divorced, separated, married/de facto). A series of single items also assessed current or past issue with gambling activities (number games, electronic gaming machines, informal private betting, table games, race betting, sports betting, and other) and current preferred gambling modality (venue, online, or both). Gambling-related harms experienced over the past year were assessed using the Domain General Harms Scale (DGHS-7: Syvertsen et al., 2023). The DGHS-7 comprises seven items measuring distinct harm domains (financial, relationship, emotional, health, work/study, cultural, legal) on a 5-point scale from 0 (no impact) to 4 (major impact). The DGHS-7 has demonstrated strong internal consistency in prior research (α = 0.88; Syvertsen et al., 2023) and this study (α = 0.85).
Reasons for early treatment disengagement	At pre-treatment, perceived barriers to attending sessions were assessed using top-loading items from three subscales of the Barriers to Retention Scale (BRS: Zemore et al., 2021) including low perceived need for treatment, fear of losing important relationships, and concerns about missing gambling. All six items from a fourth subscale were also included to assess personal limitations (3 items; e.g., forgetfulness) and logistical issues (3 items; e.g., transport). Items were rated on a 7-point scale from 1 (strongly disagree) to 7 (strongly agree), with higher scores indicating greater perceived barriers. The BRS has demonstrated good internal consistency in prior research (α = 0.88; Zemore et al., 2021) and this study (α = 0.83). At follow-up, **c**lients classified in the early disengagement group (because they reported no upcoming sessions despite not fulfilling allocated sessions or goals) were administered the 28-item Reasons for Leaving Treatment Questionnaire (RLTQ) to assess seven reasons relating to: motivational inconsistencies, staff conflicts, boundary concerns, program expectations, problem severity, and logistical problems (Ball et al., 2006). Each reason was assessed by four yes/no items. The RLTQ has demonstrated good internal consistency scale in prior research (α = 0.84) (Ball et al., 2006) and this study (α = 0.82). Clients in the early disengagement group (because they had upcoming sessions that they did not plan to attend) were administered an open-ended item to explain why they did not plan to return to treatment.
Consequences of early treatment disengagement	
Gambling-related factors	Gambling symptom severity and urges: The 12-item Gambling Symptom Assessment Scale (G-SAS) was used to assess gambling symptom severity over the past week (Kim et al., 2009), while the first four items were analysed separately to assess gambling urges. Items are rated on a scale from 0 to 4, with higher scores indicating greater symptom severity. Total scores range from 0 to 48, with established cut-points indicating mild (8–20), moderate (21–30), severe (31–40), and extreme (41–48) severity levels. The G-SAS has demonstrated strong internal consistency in prior research (α = 0.89–0.91) (Kim et al., 2009) and this study (α = 0.93). Gambling behaviour: Two single items were used to assess the number of days gambled (frequency) and the total amount of money lost to gambling (expenditure) in the past month. Gambling cognitions: The Gambling Fallacy subscale of the Jonsson-Abbott Scale (JAS) was used to assess distorted beliefs about gambling (Jonsson et al., 2017). The subscale includes three items assessing fallacies related to money (“*My gambling is a way to make money*”), skill (e.g., “*When I win*,* it is due to my skill*”), and chance (“*If I just gamble enough*,* my gambling will pay off”*). Items are rated on a 7-point scale from 1 (strongly disagree) to 7 (strongly agree), with higher scores indicating stronger endorsement of gambling cognitions. The subscale has shown good internal consistency (α = 0.83) in prior research (Jonsson et al., 2017) and this study (α = 0.75). Gambling urge self-efficacy, importance, and priority: Three sliding-scale items were used to assess confidence in resisting gambling urges, the importance placed on limiting or stopping gambling, and the extent to which change was prioritised (Dowling et al., 2021). Items are rated on a scale from 1 to 10, with higher scores indicating greater self-efficacy, importance, or priority, respectively. These items provided brief, continuous indicators of psychological readiness and commitment to change.
Psychological and social factors	Wellbeing: The Hua Oranga framework (Kingi, 2002) was used to assess mental health and wellbeing across four domains: taha wairua (spiritual), taha hinengaro (mental), taha tinana (physical), and taha whānau (family). Each domain was assessed using four items rated on a 5-point scale from 1 (strongly disagree) to 5 (strongly agree), with higher scores indicating greater wellbeing. Total and subscale scores were calculated to provide overall and domain-specific indicators of wellbeing. Although internal reliability data is limited, the Hua Oranga is widely recognised for its strong cultural relevance in New Zealand populations (McClintock et al., 2011). Anxiety symptoms: Anxiety symptoms over the past two weeks were measured using the Generalized Anxiety Disorder 2-item scale (GAD-2) (Kroenke et al., 2007). Items are rated on a 4-point scale from 0 (not at all) to 3 (nearly every day), with a total score of 3 or more suggesting possible clinically significant anxiety (Staples et al., 2019). The GAD-2 has demonstrated good screening reliability (α = 0.81) and validity (Staples et al., 2019). The GAD-2 demonstrated strong internal consistency in this study (α = 0.88).
	Depressive symptoms: Depressive symptoms over the past two weeks were assessed using the Patient Health Questionnaire-2 item scale (PHQ-2) (Kroenke et al., 2003). Items are rated on a 4-point scale from 0 (not at all) to 3 (nearly every day), with a total score of 3 or more suggesting possible clinically significant depression (Staples et al., 2019). The PHQ-2 has demonstrated good screening reliability (α = 0.83) and validity (Staples et al., 2019). The PHQ-2 demonstrated strong internal consistency in this study (α = 0.82). Risky alcohol use: A single-item screener from the Alcohol Use Disorders Identification Test (AUDIT-3) was used to assess the frequency of heavy drinking episodes (consuming six or more drinks on one occasion) (Babor et al., 2001). Responses range from 0 (never) to 4 (daily or almost daily), with higher scores indicating riskier drinking. This item has been validated as a reliable standalone screener for hazardous alcohol use (Gual et al., 2002).
	Substance use: Substance use was assessed using a validated single-item screener, which asked clients how many times they had used an illegal drug or taken prescription medication for non-medical reasons over the past month (P. C. Smith et al. 2010a, b). Impulsivity: Past-month impulsivity was assessed using six items from the State Impulsivity Scale (SIS) (Iribarren et al., 2011) comprising the two highest-loading items from each subscale: Reward (difficulty delaying immediate gratification), Automatism (repetitive behaviour despite lack of reward), and Attentional (acting quickly without adequate information). Items are rated on a 4-point scale from 1 (almost never) to 4 (almost always/always), with higher scores indicating greater impulsivity. The SIS has demonstrated strong internal reliability (α = 0.88) in prior research (Iribarren et al., 2011) and this study (α = 0.89).
	Coping strategies: Eight subscales of the Brief COPE (Carver, 1997) were used to assess dispositional coping strategies, including helpful strategies (self-distraction, active coping, positive reframing, and planning) and unhelpful strategies (self-blame and behavioural disengagement). Each subscale consists of two items rated on a 4-point scale, with higher scores indicating greater strategy use. The Brief COPE has demonstrated good internal reliability (α = 0.54–0.91.54.91) across different populations (Carver, 1997; Hagan et al., 2017) and in this study (α = 0.83). Family violence victimisation/survival and perpetration: Family violence victimisation/survival and perpetration were assessed using a modified version of the Hurt-Insult-Threaten-Scream (HITS) scale (Shirzadi et al., 2020), comprising two single yes/no items. Clients were asked whether they had used or experienced any form of emotional or physical violence in the past month by a family member.
Help-seeking behaviour	A series of single yes/no items assessed the use of nine different support options for gambling in the past month (Rodda et al., 2018). These included (1) mental health professional or residential facility for gambling, (2) mental health professional or residential facility for other mental health issues, (3) another gambling support service such as email counselling or helpline, (4) land-based exclusion (5) online exclusion (6) blocking software, family members or friends (8) peer support, and (9) psychotropic medication.
**Practitioner survey measures**	
Reasons for early treatment disengagement	Practitioners described perceived reasons through an open-ended item and rated how often they encountered 12 potential reasons using an adapted RLTQ (Ball et al., 2006). Items were updated to reflect recent review findings (Hawker et al. 2025a, b, c) on common reasons, such as logistical barriers and hopelessness. The original true/false format was replaced with a 5-point scale from 1 (not at all often) to 5 (extremely often), with higher scores indicating reasons encountered more frequently.
Consequences of early treatment disengagement	A series of five open-ended items asked practitioners to identify positive and/or negative consequences of pulling out of treatment on the client’s gambling behaviour and issues, mental health and other addictive issues, social/family support and issues, engagement in future treatment, and any other consequences. Positive consequences were conceptualised as perceived gains of even limited treatment engagement.
Solutions to improve gambling treatment retention	Two open-ended items asked practitioners to identify their top five strategies (with at least two required) that practitioners or services could use to improve treatment attendance, and any additional comments about potential solutions.

**Table 7:** Client-reported consequences of early treatment disengagement: significant correlations between percentage change in outcome from pre-treatment to follow-up and treatment status (early disengagement vs. completed/still in treatment) indicating that those who disengaged early made less gains across multiple outcomes

	Early disengagement (*n* = 11)		Completed/still in treatment (*n* = 56)		% change
	M (SD)		M (SD)		*r*
	Pre-treatment	Follow-up	Pre-treatment	Follow-up	
**Gambling-related factors**					
Gambling symptom severity (G-SAS)	**28.0 (10.9)**	**29.9 (13.8)**	**25.7 (11.5)**	**15.8 (11.7)**	**− 0.40***
Gambling urges (G-SAS subscale)	**9.6 (3.8)**	**10.4 (4.7)**	**8.4 (4.4)**	**5.0 (4.1)**	**− 0.37***
Past-month gambling frequency (days)	**13.0 (8.9)**	**11.2 (10.7)**	**11.0 (9.0)**	**4.9 (6.9)**	**− 0.26***
Past-month gambling expenditure ($NZD lost)	1585.46 (1319.06)	1243.55 (1230.88)	1425.18 (1679.50)	462.52 (706.25)	− 0.22
Gambling-related erroneous cognitions (JAS)					
Money-related	4.8 (2.1)	4.6 (2.2)	4.2 (2.0)	3.3 (1.7)	− 0.12
Skill-related	2.6 (1.7)	2.9 (1.8)	2.8 (1.8)	2.5 (1.5)	− 0.07
Chance-related	4.4 (2.2)	3.9 (1.9)	3.9 (2.0)	3.0 (1.6)	− 0.13
Gambling urge self-efficacy	**4.7 (2.4)**	**3.8 (2.9)**	**5.0 (2.6)**	**6.5 (2.6)**	**0.30***
Perceived importance of limiting gambling	8.5 (2.1)	8.5 (1.9)	8.1 (2.6)	7.9 (2.8)	0.00
Perceived priority level of limiting gambling	7.2 (3.0)	7.4 (3.1)	7.1 (2.9)	7.9 (2.6)	0.16
**Psychological and social factors**					
Overall wellbeing (Hua Oranga)	46.8 (12.6)	45.1 (14.2)	51.8 (12.8)	56.0 (13.4)	0.19
Physical wellbeing	14.9 (3.5)	13.2 (2.4)	14.7 (3.8)	15.0 (3.4)	0.24
Spiritual wellbeing	10.0 (3.6)	10.2 (4.8)	11.6 (4.3)	12.9 (4.0)	0.08
Emotional wellbeing	10.9 (2.8)	10.8 (3.9)	12.6 (3.7)	14.2 (3.8)	0.19
Family wellbeing	11.0 (3.8)	10.9 (4.2)	12.8 (3.6)	13.9 (3.8)	0.12
Anxiety symptoms (GAD-2)	**3.6 (1.2)**	**4.3 (1.6)**	**3.4 (2.0)**	**2.3 (1.9)**	**− 0.36***
Depressive symptoms (PHQ-2)	**3.2 (1.5)**	**3.7 (1.4)**	**2.9 (1.9)**	**1.8 (1.6)**	**− 0.38***
Risky alcohol use (AUDIT-3)	**1.9 (1.8)**	**2.3 (1.6)**	**1.4 (1.3)**	**0.8 (1.0)**	**− 0.25***
Past-month substance use (number of times)	**7.0 (7.9)**	**15.6 (28.7)**	**2.8 (5.7)**	**2.2 (4.4)**	**− 0.26***
Past-month experience of family violence (yes/no)					
Victim/survivor of family violence	0.1 (0.3)	0.2 (0.4)	0.2 (0.4)	0.1 (0.3)	− 0.17
Perpetrator of family violence	0.1 (0.3)	0.2 (0.4)	0.1 (0.3)	0.0 (0.1)	− 0.18
Impulsivity (SIS)	8.4 (3.0)	7.6 (3.6)	6.7 (4.7)	5.0 (3.6)	− 0.08
Reward	2.7 (1.5)	2.5 (1.5)	2.2 (1.7)	1.6 (1.3)	− 0.05
Automatism	2.3 (1.6)	2.5 (1.5)	2.0 (1.7)	1.4 (1.4)	− 0.16
Attentional	3.4 (1.4)	2.7 (1.3)	2.5 (1.9)	2.0 (1.7)	0.01
Coping behaviour (Brief COPE)					
Self-distraction	5.5 (1.8)	7.2 (2.6)	4.8 (1.7)	6.5 (1.9)	0.07
Active coping	5.3 (1.5)	5.2 (1.7)	5.3 (1.5)	6.4 (1.9)	0.18
Use of emotional support	4.4 (1.7)	6.7 (2.5)	4.9 (1.9)	6.5 (2.2)	− 0.08
Use of instrumental support	4.7 (1.7)	6.9 (1.9)	4.8 (1.8)	6.7 (2.0)	− 0.05
Positive reframing	4.5 (1.7)	6.8 (1.4)	4.8 (1.6)	6.3 (2.2)	− 0.17
Self-blame	6.2 (1.5)	5.1 (2.2)	6.1 (2.0)	5.1 (1.9)	0.02
Planning	5.5 (1.8)	5.9 (2.5)	5.5 (1.7)	5.9 (2.1)	0.02
Behavioural disengagement	4.3 (1.7)	6.2 (2.9)	4.1 (1.9)	6.2 (2.3)	0.05
**Help-seeking behaviour**					
Past-month help-seeking (yes/no)					
Mental health professional or residential gambling facility	0.5 (0.5)	0.4 (0.5)	0.6 (0.5)	0.4 (0.5)	− 0.01
Mental health professional or residential facility for mental health issue other than gambling	**0.3 (0.5)**	**0.5 (0.5)**	**0.6 (0.5)**	**0.4 (0.5)**	**− 0.25***
Another support service for gambling (e.g., helpline, email counselling)	0.6 (0.5)	0.4 (0.5)	0.6 (0.5)	0.4 (0.5)	0.11
Exclusion from land-based venue	0.2 (0.4)	0.2 (0.4)	0.3 (0.4)	0.2 (0.4)	− 0.05
Exclusion from online gambling site/app	0.5 (0.5)	0.5 (0.5)	0.4 (0.5)	0.4 (0.5)	− 0.16
Online blocking software	0.4 (0.5)	0.3 (0.5)	0.3 (0.5)	0.3 (0.5)	0.08
Friend and family members	0.8 (0.4)	0.5 (0.5)	0.6 (0.5)	0.6 (0.5)	0.19
Peer support	0.4 (0.5)	0.3 (0.5)	0.4 (0.5)	0.3 (0.5)	0.04
Psychotropic medication use	0.4 (0.5)	0.3 (0.5)	0.3 (0.5)	0.2 (0.4)	0.01

AUDIT-3 = Alcohol Use Disorders Identification Test; G-SAS=Gambling Symptom Assessment Scale; GAD-2 = Generalized Anxiety Disorder-2; JAS=Jonsson-Abbott Scale; NZD = New Zealand Dollars; PHQ-2 = Patient Health Questionnaire-2; SIS=State Impulsivity Scale. Higher scores on the: GSAS indicate greater gambling symptom severity; JAS indicate stronger erroneous cognitions; Hua Oranga indicate greater wellbeing; GAD-2 indicate greater anxiety symptoms; PHQ-2 indicate greater depressive symptoms; AUDIT-3 indicate more frequent risky drinking; SIS indicate greater impulsivity; and Brief COPE indicate greater use of the respective coping behaviour. In Spearman correlations, the ‘*early disengagement’* group (*n* = 11) was coded as 0 and the combined ‘*completed/still in treatment’* group (*n* = 56) was coded as 1. Correlation strength was interpreted as negligible (rs= 0.00 to ± 0.20), weak (± 0.21 to ± 0.40), moderate (± 0.41 to ± 0.60), strong (± 0.61 to 0.80), and very strong (± 0.81 to ± 1.00). ***Significant at** ***p*** **<.05**
